# Throughput Maximization for Sensor-Aided Cognitive Radio Networks with Continuous Energy Arrivals

**DOI:** 10.3390/s151229766

**Published:** 2015-11-27

**Authors:** Thanh-Tung Nguyen, Insoo Koo

**Affiliations:** Department of Electrical/Electronics and Computer Engineering, University of Ulsan, 93-Daehak-ro, Nam-gu, Ulsan 680-749, Korea; nguyenthanhtung@ctu.edu.vn

**Keywords:** sensor network, sensor scheduling, energy harvesting, cooperative spectrum sensing, cognitive radio network

## Abstract

We consider a Sensor-Aided Cognitive Radio Network (SACRN) in which sensors capable of harvesting energy are distributed throughout the network to support secondary transmitters for sensing licensed channels in order to improve both energy and spectral efficiency. Harvesting ambient energy is one of the most promising solutions to mitigate energy deficiency, prolong device lifetime, and partly reduce the battery size of devices. So far, many works related to SACRN have considered single secondary users capable of harvesting energy in whole slot as well as short-term throughput. In the paper, we consider two types of energy harvesting sensor nodes (EHSN): Type-I sensor nodes will harvest ambient energy in whole slot duration, whereas type-II sensor nodes will only harvest energy after carrying out spectrum sensing. In the paper, we also investigate long-term throughput in the scheduling window, and formulate the throughput maximization problem by considering energy-neutral operation conditions of type-I and -II sensors and the target detection probability. Through simulations, it is shown that the sensing energy consumption of all sensor nodes can be efficiently managed with the proposed scheme to achieve optimal long-term throughput in the window.

## 1. Introduction

Cognitive radio (CR) technology and green wireless systems have received much attention from the radio research community as promising approaches for improving spectrum utilization and enhancing energy efficiency, respectively. In wireless communications, the demand for broadband wireless spectrum is increasing. Due to the increase in personal wireless mobile devices with a growing number of online interactive applications embedded in them, such as multimedia services, gaming, *etc.*, mobile devices need a broadband wireless spectrum to transfer more data for higher quality of service. There seems to be a serious problem due to the scarcity of the spectrum, but according to the FCC [1,2], many frequency bands are under-utilized in temporal and spatial dimensions while others are heavily used. Hence, ineffective spectrum access is the reason for such problems rather than physical scarcity of the spectrum. In this context, in 1999 in his dissertation Mitola first proposed CR technology was to cope with the urgent problem of spectrum scarcity. The proposal is of interest to the radio research community because unlicensed/secondary users (SUs) or cognitive radio users (CUs) can be allowed to exploit under-utilized licensed radio channels simultaneously used by licensed/primary users (PUs) in order to improve the channel performance while avoiding interference with the PUs.

Along with ineffective usage of spectrum resources, saving energy consumption for mobile devices is also realized as a problem and becomes a key objective in the design of both energy-efficient future hardware and green radio communications [3]. Future radio hardware designs must also cope with the utilization of renewable energy harvesting sources from the ambient environment such as in the cases of solar, wind, vibration, thermal source, and radio power transmission. Most of the research done on energy harvesting in the context of cognitive radio [4,5,6,7,8,9] has considered different scenarios of single secondary user settings with an energy harvester. In [4], Sultan investigated access policies for only a single SU with an energy harvester [4]. At the beginning of each timeslot, based on available energy and possibilities of arrival harvested energy packets, an MDP framework is utilized to determine which action to perform, spectrum sensing, transmission, or sleeping, to maximize long-term throughput. In one study, the problem of maximizing the short-term throughput of an energy harvesting node was investigated [5]. The authors investigated two related problems pertaining to short-term throughput maximization and the minimization of transmission completion time. The optimum transmission policies are identified under constraints on energy causality and replenishment. A model with discrete packets of energy arrivals is considered. The goal is to find optimum power allocation for maximizing the total data transferred from an energy harvesting node under a deadline constraint. In another study, a secondary user capable of harvesting energy is considered [6]. This work states that sensing threshold affects the probability of accessing the idle/occupied spectrum of the secondary user and that the secondary user can operate in two modes, the spectrum-limited regime or the energy-limited regime, depending on the energy arrival rate and the expected energy consumption for sensing and spectrum access. Given a fixed sensing duration, this work determines an optimal sensing threshold to maximize the number of successful spectrum accesses in order to maximize expected total throughput of the secondary user. Especially, an extended version of this work [6] is investigated in a different study [7]. Throughput maximization for a single secondary user with energy harvesting function is also considered where a pair of sensing threshold and sensing duration is fully studied. The work aims to find both the optimal sensing threshold and the optimal sensing duration for the secondary user such that the shorter sensing duration which still satisfies the collision constraint is preferred for saving energy consumption. This positively affects expected throughput of the secondary user. In [8], Chen *et al.* considered an information transmitter and aims to find an optimal tradeoff between harvesting RF energy from a power source in the first phase and utilized the harvested energy for transferring information to an information receiver in the second phase such that average information transmission rate is improved. In [9], Zhang *et al.* considered a system in which information is transferred from a base station to an information-decoding user and is also eavesdropped by an energy harvesting use when the transmitter and receivers are equipped with multi-antenna. The objective is to determine the optimal transmit direction of antennas in beam-forming scheme at the base station for maximizing secrecy rate while the harvested energy requirement for the energy harvesting user is satisfied.

In [10], Sensor-Aided Cognitive Radio Network (SACRN) in which sensors are completely powered by batteries and distributed over the network was considered to support cognitive radio networks in detecting the activity of the PU network. SACRN is now considered one of the most appealing approaches to perform cost-effective spectrum sensing in CR. The base station of cognitive radio network examined sensing the results of each sensor and the base station divided sensors into several non-disjoint groups of sensors where global probability of detection is satisfied in each group. The objective is to schedule on/off of each sensor group for cooperative spectrum sensing to detect the absence/presence of a primary user in an energy efficiency manner such that it prolongs the sensor network lifetime. In [11], Liu *et al.* developed a group-based cooperative MAC protocol (GC-MAC) for selecting multiple SUs to form several teams of SUs where SUs in each team are cooperative in spectrum sensing for one distinct channel until at least one available channel is found. The tradeoff between sensing accuracy and efficiency for two types of channel condition: time-invariant channel and time-varying channel were investigated. In [10,11], energy harvesting is not considered and further only short-term throughput is considered.

In the paper, we consider a SACRN in which sensor nodes capable of harvesting energy are distributed over CR network to support secondary transmitters in sensing the licensed channel. Unlike previous papers related to SACRN [10,11], we investigate the maximization of long-term throughput of SACRN by considering the scheduling window composed of multiple time slots as well as continuous energy arrival rate. Further, two types of energy harvesting sensor nodes (EHSN) are considered where type-I sensor nodes will harvest ambient energy in whole slot duration, whereas type-II sensor nodes will only harvest energy after carrying out spectrum sensing. The objective of the paper is to schedule harvested-energies of different sensor nodes for Cooperative Spectrum Sensing (CSS) over the scheduling window to detect availability of licensed channel for SU access as well as to maximize long-term throughput under the constraints of energy-neutral operation and primary user collision. The energy-neutral operation is to guarantee that the energy consumption must be less than the sum of available energy and energy harvested in a particular amount of time, whereas the collision constraint needs to be satisfied to protect primary users from licensed channel access of secondary users.

The remainder of this paper is organized as follows: in Section 2, we describe a system model in which a primary network, a cognitive radio network, and a sensor network are the main components In Section 3, we describe the energy constraints of two types of sensors for spectrum sensing. In Section 4, we formulate the throughput maximization problem by considering energy-neutral operation conditions of type-I and -II sensors and the target detection probability, and further describe the numerical solving method. In Section 5, three simulation experiments are presented to show the effectiveness of the proposed scheme. In Section 6, we draw the conclusions.

## 2. Energy Harvesting Sensor-Aided Network Model

**Figure 1:**
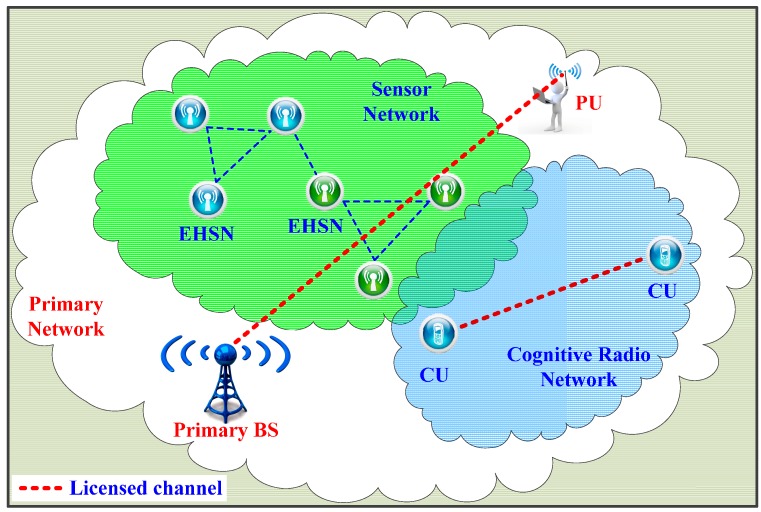
Sensor-Aided Cognitive Radio Network (SACRN).

In this section, we will describe different aspects of the operation of a primary network and energy harvesting SACRN. Generally, the sensor network architecture comprises three parts, as shown in Figure 1, where the sensor network is deployed on a primary network and performs spectrum sensing, whereas cognitive radio network requests the sensor network for sensing licensed channels to perceive the channel state.

### 2.1. Primary Network Model

We consider a simple primary network setting, as shown in Figure 1, where a licensed channel with a bandwidth *W* operates in a time-slotted fashion with equal slot durations. In previous works, a licensed channel is usually modeled as Markov chain with two states: The idle state and the busy state [4]. Transition probabilities between two states are assumed to be known such that a secondary user can track the channel state for a spectrum sensing strategy and decide to sense the channel or not based on available energy at the beginning of current timeslot. However, in this paper, we simply assume that the probability of an idle channel at each timeslot can be measured by the cooperation between the sensor network and the cognitive radio network in spectrum sensing and channel access, respectively. We denote *I* ∈ {0 (*idle*), 1 (*occupied*)} as the action set of the primary user on the licensed channel and define *P*_0_ = Pr {*I* = 0} as the probability that the licensed channel is not occupied by the primary user and is available for a SU utilization.

### 2.2. Cognitive Radio Network Model

In Cognitive Radio Network (CRN), as shown in Figure 1, we consider a link established on the licensed channel between two secondary users and assume that data is always available to be transferred from the secondary transmitter to the secondary receiver. Based on sensing results periodically reported from the sensor network, the secondary transmitter will determine whether or not to send data to the secondary receiver over the link. Whenever a report of an idle channel is sent from the sensor network, there exist two possibilities of data transmission. In the case that the licensed channel is actually free under hypothesis *H*_0_, the secondary transmitter sends data to the secondary receiver successfully. In this case, the throughput obtained in the whole slot duration over the secondary link can be approximated and is given by *C*_0_ = *W*log(1 + γ), where γ refers to the signal-to-noise ratio (SNR) of the transmitting signal measured at the secondary receiver. In practice, however, the amount of transferred data is less than *C*_0_ due to the sensing time before data transmission. Thus, less sensing time is an important control parameter for optimal throughput. In the simulation, we only study the throughput with a designed value of *C*_0_ without further considering the SNR at the secondary receiver. In the second case of data transmission, the channel is misunderstood as in an idle state under hypothesis *H*_1_ due to an incorrect decision from the sensor network. In this case, the secondary transmitter fails to send its data to the secondary receiver for an interference collision with an existing primary user on the channel.

### 2.3. Sensor Network Model

Much recent research states that a single sensor node cannot reliably detect the presence/absence of a primary user due to the effect of multipath fading or shadowing in the transmission environment, which is a crucial issue in spectrum sensing. Cooperation among sensor nodes in spectrum sensing greatly enhances the probability of detection due to the diversity of multiple sensor nodes. For this reason, as with the reference [10] in which sensor nodes powered by a battery are considered, we investigate a sensor network (SN) deploying multiple EHSNs where all sensor nodes are not only powered by batteries but also have the capability of harvesting energy for self-operation over time. Due to being in different locations in the network, different EHSNs receive different signal-to-noise (SNR) from the primary user and harvest different amounts of energy from the environment.

Furthermore, cluster formation and scheduling can be utilized to increase network lifetime of sensor neworks [12,13] where sensor nodes are grouped into non-disjoint clusters, and each cluster is activated successively to extend the network lifetime. For cluster formation, the residual energy of sensor nodes and SNR to the primary channel can be taken into account such that minimum energy level and minimum SNR of sensor nodes of each cluster can be guaranteed for performing spectrum sensing. Therefore, in the paper we divide the whole sensor network into clusters (cluster formation) and update the clusters if needed. In the cluster formation, the residual energy of sensor nodes and SNR to the primary channel are also taken into account to guarantee minimum sensing performance of each cluster. That is, only sensors that satisfy minimum energy level and minimum SNR are grouped in to clusters by the FC. Therefore, we can assume that minimum energy level and minimum SNR of sensor nodes of each cluster are guaranteed for each sensor to participate in spectrum sensing throughput cluster formation. After SN is decomposed into disjoint clusters, activating only one cluster, instead of all clusters in a SN, significantly reduces energy consumption because deactivated clusters are switched to sleep mode. It is noteworthy that the objective of this paper is to investigate long-term throughput of each active cluster for given scheduling window, and formulate the throughput maximization problem by considering energy-neutral operation conditions of two types of sensors and the target detection probability.

In addition, the amount of energy consumption must follow some constraints for optimal performance in CSS of each cluster. Correspondingly, CSS among EHSNs in a cluster under the initial energy, distributed amount of energy harvesting, and constraints of energy consumption need to be examined and investigated for long-term throughput of the CRN. Energy scheduling is performed by the FC of the SN, and the designed sensing time for each EHSN in a cluster is therefore assigned before the beginning of spectrum sensing with objective to satisfy the collision threshold constraint and to achieve optimal long-term throughput of the CRN. For each cluster, the FC will control distributed EHSNs in spectrum sensing in an optimal way and frequently report global decisions to the CRN to serve the channel accesses of a pair of secondary users. In this paper, we assume that each cluster of SN is consisted of *V* EHSNs distributed over the network for exploiting the opportunities of accessing the licensed channel accesses of the secondary users.

## 3. Energy-Constraint Operation

This section investigates sensing operation of EHSNs in a cluster under energy constraints based on initial energy, energy harvested during the sensing duration and transmission, and remaining energy. The aim is to determine how much energy each EHSN should spend for CSS in designed timeslots.

### 3.1. Spectrum Sensing

Spectrum sensing plays a crucial role in the procedure of licensed channel access of any SU. In overlay mode, before a SU accesses the licensed channel, a sensor node senses the spectrum for a duration referring to sensing time and denoted by τ ∈ [0, *T*] where *T* is the slot duration. Two important parameters associated with spectrum sensing results are probability of detection and probability of false alarm, which are the probability that a sensor can detect the presence of a primary user under hypothesis *H*_1_ and the probability that a sensor does not detect an idle licensed channel under hypothesis *H*_0_, respectively. On the one hand, from the PU perspective, spectrum sensing protects PU from collisions in opportunistic channel access of SUs. A good system should have a high probability of detection to reduce collisions between the SU and the PU. For instance, if a higher probability of detection is guaranteed, the primary users are protected better. On the other hand, in the SU perspective, spectrum sensing offers valuable chances to utilize licensed channels, such that a lower probability of false alarm is obtained, and there is a higher chance that SUs can successfully access a licensed channel in the case of PU absence. Moreover, sensing time has tremendous impacts on two sensing parameters. For example, in the case that a sensor node spends more time for spectrum sensing, the probability of detection becomes higher and the probability of false alarm becomes smaller. However, more sensing time means that SUs have less time for data transmission, and that more energy is consumed. It is clearly that time spent for sensing is limited by the amount of available energy in battery. In this paper, we assume that sensing energy is linearly related to sensing time, such that *e_s_* = τ*P_s_* where *P_s_* = ℜ^+^ is the sensing power of sensor nodes.

As previously mentioned, licensed channels are organized in a timeslot structure with equal slot durations. To exploit the licensed channel, EHSNs in a cluster perform CSS before SUs transmit data. Energy consumption due to the sensing operation needs to satisfy an energy causality constraint which is mentioned in the next subsection. Let us assume that a sensor node *v* in the cluster carries out spectrum sensing on a licensed channel at timeslot *s*, and denote $P_{vs}^{d}$ and $P_{vs}^{f}$ as the local probability of detection and the local probability of false alarm extracted from the spectrum sensing results of sensor *v* at timeslot *s* respectively. In this case, the local probability of detection and the local probability of false alarm are given as in a previous study [14]:

(1) $$P_{vs}^{d}=Q\left\{\left(x_{vs}-\delta_{v}\sqrt{\tau_{vs}}\right)\xi_{v}^{-1}\right\}$$

(2) $$P_{vs}^{f}=Q\left\{\xi_{v}y_{vs}+\delta_{v}\sqrt{\tau_{vs}}\right\}$$

where Q(x)=12π∫x∞e−t22dt is the complementary distribution function of a standard Gaussian random variable. $x_{vs}=Q^{-1}(P_{vs}^{f})$ and $y_{vs}=Q^{-1}(P_{vs}^{d})$ are inverse translations of the local probability of false alarm and the local probability of detection, respectively. $\xi_{v}=\sqrt{2\gamma_{v}+1}$, $\delta_{v}=\gamma_{v}\sqrt{f_{s}}$, γ_*v*_ indicates the SNR presented at sensor *v* and *f_s_* is the sampling frequency (*ξ_v_* > 0, *δ_v_* > 0). τ_*vs*_ is the sensing time of the sensor *v* on the licensed channel at timeslot *s*. In Equations (1) and (2), given SNR, if we fix either probability of detection or probability of false alarm and vary sensing time, we can find probability of false alarm or probability of detection, respectively.

From Equations (1) and (2), the sensing time τ_*vs*_ of sensor *v* at timeslot *s* with respect to the local probability of detection and the local probability of false alarm can be derived as follows [15]:

(3) $$\tau_{vs}={\left\{\left(x_{vs}-y_{vs}\xi_{v}\right)\delta_{v}^{-1}\right\}}^{2}$$

From Equation (3), it can be inferred that, for the energy issue, we first design the local probability of detection and the local probability of false alarm in order to determine a corresponding sensing time. Therefore, sensing energy, in this case, can be computed.

We assume that FC uses the “OR” rule in making a global decision in the presence/absence of a PU. In this rule, FC determines timeslot *s* as free if all sensor nodes report the same result that a PU is absent from the channel (*I* = 0) to the FC. In this case, the global probability of detection and the global probability of false alarm at the timeslot *s* are respectively given by:

(4) $$Q_{s}^{D}=1-\prod_{v\in V}\left(1-P_{vs}^{d}\right)=1-\prod_{v\in V}\left\{1-Q(y_{vs})\right\}$$

(5) $$Q_{s}^{F}=1-\prod_{v\in V}\left(1-P_{vs}^{f}\right)=1-\prod_{v\in V}\left\{1-Q(x_{vs})\right\}$$

### 3.2. Scheduling Window

**Figure 2:**
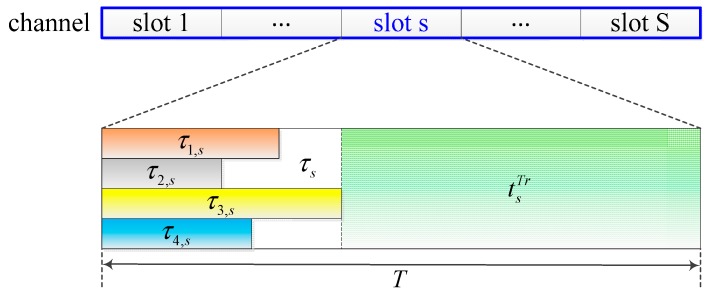
Structure of scheduling window and timeslot where scheduling window is consisted of *S* time slots when four sensor nodes exist. τ_*is*_ is sensing time of the ith sensor node where *i* = 1, …, 4, and $t_{s}^{Tr}$ is transmission time of SU at time slot *s*.

In an activating cluster, based on available energy at beginning of a timeslot and the capability of harvesting energy of each EHSN in the timeslot, FC will calculate how much time each EHSN should spend for CSS to maximize achievable throughput in the timeslot [10,11]. However, more than one timeslot can be considered to efficiently manage energy consumption of all sensor nodes in the cluster and further to get more throughput. Therefore, in the paper we consider the scheduling window for more efficient energy consumption of sensor nodes. Figure 2 shows an example for the scheduling window that is consisted of *S* time slot when four sensor nodes exist. Here, let us define *S* as the window size (WS). For the scheduling window, energy operation of EHSNs will be managed more effectively by the proposed scheme.

### 3.3. Evaluation Method

In this paper, we evaluate the spectrum sensing performance of the considered cluster through achievable throughput of SU. In overlay mode, a SU transmitter can only send its data over a licensed channel after a CSS among EHSNs is completed and the channel is determined to be free. Transmission time of SU in timeslot *s* is mainly affected by the sensing duration of EHSNs, and can be expressed as:

(6) $$t_{s}^{Tr}=T-\tau_{s}=T-max\left(\tau_{vs}+t_{vs}^{r}\right)$$

where $t_{vs}^{r}$ is the reporting time of sensor *v* on a licensed channel at timeslot *s*, and τ_*s*_ is a period of time at the beginning of timeslot *s* in which all EHSNs must finish sensing the channel and report sensing results to FC, as shown in Figure 2. In this paper, the reporting time is assumed to be fixed.

Based on the mentioned scheduling window, we now denote *R_s_* as achievable throughput of SU in timeslot *s*. The throughput approximation can be calculated by:

(7) $$R_{s}=P_{0}\left(1-Q_{s}^{F}\right)\frac{T-\tau_{s}}{T}C_{0}$$

where *C*_0_ is maximal throughput which is mentioned in Section 2.2. Under hypothesis *H*_0_, the expression $1-Q_{s}^{F}$ represents the probability that SU can access a licensed channel successfully. The notation *P*_0_ represents the probability that a timeslot *s* is free on a licensed channel, which depends on traffic of the PU on the considering channel. It is clear that achievable throughput is affected by three factors: sensing capability of EHSNs in SN, channel condition that SU suffers from in SACRN, and channel utilization of the PU in primary network. In this paper, we try to impact the first mentioned factor in the best way.

Basically, the main problem is to maximize achievable throughput of SACRN in a designed scheduling window, which can be expressed as follows:

(8) $$max\;R=\sum_{s=1}^{S}R_{s}$$

(9) $$s.t.\;\;\;Q_{s}^{D}\geq Q_{th}^{d}$$

The constraint expressed by Equation (9) guarantees that the global probability of detection must be greater than a desired threshold in every timeslot of the scheduling window.

### 3.4. Energy Harvesting Operation

Energy efficiency can be evaluated based on initial energy and remaining energy both in each timeslot and in their whole scheduling window. Thus, in this subsection, we first describe the initial energy and energy harvesting operation of EHSNs in the considered cluster, and then consider energy consumption of EHSNs along with energy constraint settings.

#### 3.4.1. Energy-Harvesting Model

Although batteries and their corresponding capacities are modeled in different ways in existing works, such as ideal/imperfect battery with a finite/infinite capacity, throughout this paper we merely consider sensor nodes outfitted with an ideal battery having an infinite capacity. Specifically, each sensor node is equipped with an energy harvester. Such sensors will gather continuous flows of ambient energy from sources of continuous energy arrival fashion.

It is assumed that instantaneous energy arrival rate at time instant *t*, denoted by *g*(*t*), is non-negative and integrable where *g*(*t*) ∈ [*g_min_*, *g_max_*] and *g_min_* > 0. Generally, the total energy of an EHSN at time instant *t* comprises the available energy *e*_0_ in battery at the beginning of a timeslot and the energy harvested by time *t* [16] as following:

(10) $$e(t)=e_{0}+\int_{0}^{t}g(\tau )d\tau$$

**Figure 3:**
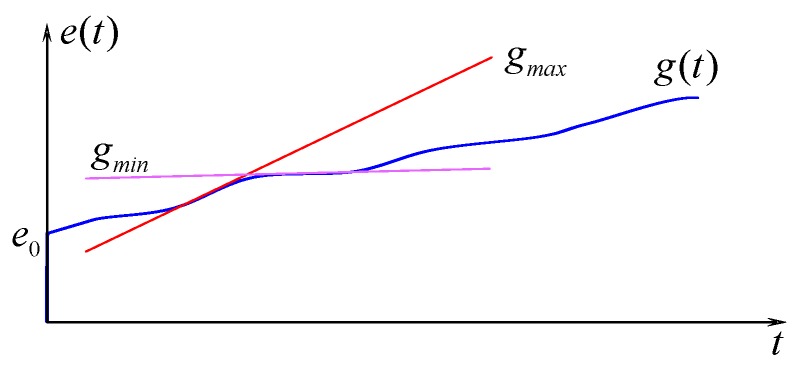
Example of energy harvesting rate and harvested energy by time.

Obviously, the total energy is non-decreasing by time and mainly depends on instantaneous energy arrival rate *g*(*t*) which varies in specific ranges as depicted in Figure 3. In the paper, we assume that the energy arrival rate *g*(*t*) varies very gently and slowly between *g_min_* and *g_max_* per window not time slot. Therefore, the value of *g*(*t*) is calculated at the beginning time of each window and will be maintained as constant for that window. At the beginning time of next window, the value of *g*(*t*) will be calculated again, and be maintained as constant for in the window, and so on. In the paper, we use *g_min_* for scheduling the total energy for sensing operations among timeslots in a scheduling window. Throughout the paper, the lower bound of instantaneous energy arrival rate is also used as the energy harvesting rate of sensor node. For this assumption, the minimum cumulative amount of energy at time instant *t* can be simplified as follows:

(11) $$e_{min}^{h}(t)=e_{0}+g_{min}t$$

#### 3.4.2. Type of Sensors

In a practical sensor network, each sensor node may have a typical type of energy harvester depending on energy source, such as thermal source, solar, vibration, *etc*. In the paper, we consider two types of EHSN: Type-I sensor nodes can harvest ambient energy in the whole slot duration. On the other hand, type-II sensor nodes can only harvest energy after carrying out spectrum sensing. That is, type-II sensor nodes cannot harvest energy during spectrum sensing.

Let us assume that the activating cluster of SN comprises *N* type-I sensor nodes and *M* type-II sensor nodes, and the number of sensor nodes in the cluster therefore will be *V* = *N* + *M*.

Generally, let $e_{vs}^{h}\in {ℜ}^{+}$ denote the amount of harvested energy of a sensor *v* of the cluster at timeslot *s*, where *v* ∈ {*n*, *m*}. Specially, depending on energy harvesting rate *g_v_*(*t*), a type-I sensor node *n* of the cluster can harvest a constant amount of energy given by $e_{ns}^{h}=Tg_{n}$ where *n* ∈ [1, *N*] and *g_n_*(*t*) = *g_n_*, regardless of whether they are performing spectrum sensing or not. At the same time, a type-II sensor node *m* of the cluster gathers a varying amount of energy $e_{ms}^{h}=(T-\tau_{ms})g_{m}$, where *m* ∈ [1, *M*] and *g_m_*(*t*) = *g_m_*. This means that if more the sensing time τ_*ms*_ is spent, less energy is harvested. Obviously, it is necessary to measure the sensing time of a type-II sensor node in order to know how much energy it harvests. It is worth noting that type-I sensor nodes can operate in Mode II, whereas the type-II sensor nodes cannot operate in Mode I.

Now, let us further denote $e_{vs}^{s}$ as sensing energy of a sensor *v* on a licensed channel in timeslot *s*. Generally, in a scheduling window, available energy of the sensor *v* at the beginning of timeslot *s* comprises the initial energy at the first timeslot and energy which is harvested and consumed before timeslot *s*. Hence, the available energy of the sensor *v* of the cluster at timeslot *s* can be calculated as:

(12) $$e_{vs}^{a}=e_{v1}^{a}+\sum_{i=1}^{s-1}e_{vi}^{h}-\sum_{i=1}^{s-1}e_{vi}^{s}\;\;\;\forall s\in [1,S]$$

From the above expression, available energy at the beginning of timeslot *s* of a type-I sensor node *n* and of a type-II sensor node *m* is respectively expressed as follows:

(13) $$e_{ns}^{a}=e_{n1}^{a}+(s-1)Tg_{n}-\sum_{i=1}^{s-1}e_{ni}^{s}\;\;\;\forall n\in [1,N]$$

(14) $$e_{ms}^{a}=e_{m1}^{a}+(s-1)Tg_{m}-\left\{1+\frac{g_{m}}{P_{m}^{s}}\right\}\sum_{i=1}^{s-1}e_{mi}^{s}\;\;\;\forall m\in [1,M]$$

where $P_{m}^{s}$ is the sensing power of the type-II sensor node *m*.

#### 3.4.3. Energy-Neutral Operation

As in [6,7], Energy-Neutral Operation (ENO) can be defined as follows: Energy used is less than the energy harvested from the environment. To satisfy ENO, total energy consumption *e_c_*(*t*) of each sensor node at given time instant *t* must satisfy the following energy causality constraint:

(15) $$e_{c}(t)\leq e_{0}+\int_{0}^{t}g(\tau )d\tau$$

In the paper, we consider a cluster with two types of EHSN: Type-I sensor node and type-II sensor node. In the following sub-section, we will examine ENO for each type of sensor nodes to find the relationship of sensing time among timeslots in a scheduling window.

##### Energy-Neutral Operation of Type-I Sensor Node

At first, we consider ENO and investigate the energy causality constraint for type-I sensor nodes in a timeslot *s*, which can be expressed as follows:

(16) $$\int_{0}^{\tau_{ns}}P_{n}^{s}dt\leq \int_{0}^{\tau_{ns}}g_{n}dt+e_{ns}^{a}$$

This is due to the previously mentioned capability of the type-I sensor node with which it can harvest energy and perform spectrum sensing simultaneously. The constraint Equation (16) can be explained that the energy consumed due to spectrum sensing by time instant *t* cannot exceed the total of available energy in the battery at the beginning of the current timeslot and the amount of energy harvested while sensing the licensed channel by time instant *t*. By substituting Equation (13) into Equation (16), we can rewrite constraint Equation (16) as following:

(17) $$P_{n}^{s}\tau_{ns}\leq g_{n}\tau_{ns}+e_{ns}^{a}\;\Leftrightarrow \;\tau_{ns}\leq e_{ns}^{a}{\left(P_{n}^{r}\right)}^{-1}$$

where $P_{n}^{r}=P_{n}^{s}-g_{n}>0$ means that sensing power is greater than the power of energy harvesting. The right-hand quantity $e_{ns}^{a}{\left(P_{n}^{r}\right)}^{-1}$ of Equation (16) indicates the maximum possible sensing time of a type-I sensor, and it depends on available energy at the beginning of the timeslot *s*, $e_{ns}^{a}$, which is given by Equation (13) and $P_{n}^{r}$. Accordingly, the constraint Equation (16) for energy causality can be changed to a new constraint Equation (17) in terms of maximum possible sensing time in the scheduling window, which will be utilized as a constraint of throughput maximization formulation in Section 4.

Even though a type-I sensor node of the cluster has sufficient energy in the battery, the sensing time of type-I sensor cannot exceed the slot duration. Subsequently, the energy causality constraint for the type-I sensor nodes should be changed by considering the slot duration *T* such that we have:

(18) $$\tau_{ns}\leq min\left\{e_{ns}^{a}{\left(P_{n}^{r}\right)}^{-1},T\right\}$$

From Equation (18), we have two cases of energy causality constraints for the type-I sensor node follows as follows.

In the first case that $e_{ns}^{a}{\left(P_{n}^{r}\right)}^{-1}<T$, the maximum sensing time is less than slot duration. The constraint for sensing time at a timeslot *s* is affected by sensing time at previous timeslots and itself also make effect on sensing time at subsequent next timeslots. That is, the relationship among sensing times of timeslots in the scheduling window can be explained by following equation that can be obtained by substituting Equation (13) into Equation (17):

(19) $$P_{n}^{r}\tau_{ns}+P_{n}^{s}\sum_{i=1}^{s-1}\tau_{ni}-\left\{e_{n1}^{a}+(s-1)Tg_{n}\right\}<0$$

In the second case that if $e_{ns}^{a}{\left(P_{n}^{r}\right)}^{-1}\geq T$, the maximum sensing time is larger than slot duration *T*. In the case, the sensing time is limited to the slot duration, which is expressed as $\tau_{ns}-T\leq 0$. Due to abundant amount of available energy at the beginning of a timeslot *s*, the sensing time in timeslot *s* is not affected by the sensing time at previous timeslots, but it makes effect on sensing times of subsequent timeslots in the scheduling window.

Particularly for energy efficiency, we need to control the amount of remaining energy of a sensor *n* at timeslot *s*. For this purpose, we can control the amount of energy spent for spectrum sensing in the current timeslots. Now, let $e_{ns}^{r}\in {ℜ}^{+}$ denote the amount of remaining energy of a type-I sensor node *n* at the end of a timeslot *s*. Obviously, this remaining energy also directly refers to available energy for the next timeslot *s* + 1. Thus, the constraint for this case in terms of energy is expressed as follows:

(20) $$e_{ns}^{r}=e_{n(s+1)}^{a}\geq e_{ns}^{r.th}$$

where $e_{ns}^{r.th}\in {ℜ}^{+}$ represents a designed threshold of remaining energy at timeslot *s*. It is noteworthy that for each sensor node we can set up remaining energy threshold either at every timeslot *s* ∈ [1, *S*] or at the last timeslot *S* in the scheduling window only.

In terms of sensing time, the constraint for controlling remaining energy at timeslot *s* also can be expressed as follows:

(21) $$P_{n}^{s}\sum_{i=1}^{s}\tau_{ni}-\left\{\left(e_{n1}^{a}+se_{ns}^{h}\right)-e_{ns}^{r.th}\right\}<0$$

The first term in constraint Equation (21) represents the total energy consumption for sensing a licensed channel, whereas the second term is the maximum energy that the type-I sensor node can consume in the given time slot. This constraint represents the energy causality for the case of controlling energy.

##### Energy-Neutral Operation of Type-II Sensor Node

For the second case of the operation mode, energy causality and remaining energy constraints for the type-II sensor node *m* of the cluster can be similarly determined as follows:

(22) $$\int_{0}^{\tau_{ms}}P_{m}^{s}dt\leq \int_{0}^{\tau_{ms}}g_{m}dt+e_{ms}^{a}=e_{ms}^{a}$$

In this case, the type-II sensor node cannot harvest energy while performing spectrum sensing. Thus, energy harvesting rate in the sensing duration actually becomes zero, *g_m_* = 0. This type of sensor must first consume available energy in the battery for performing spectrum sensing before harvesting energy.

Combining the constraints Equations (14) and (22) gives us the energy causality constraint, which is further arranged as follows:

(23) $$P_{m}^{s}\tau_{ms}\leq e_{ms}^{a}\Leftrightarrow \tau_{ms}\leq e_{ms}^{a}{\left(P_{m}^{s}\right)}^{-1}$$

Consequently, the energy causality constraint for the type-II sensor node will become:

(24) $$\tau_{ms}\leq min\left\{e_{ms}^{a}{\left(P_{m}^{s}\right)}^{-1},T\right\}$$

Like Equation (18), two cases of energy causality constraints for the type-II sensor nodes can be further explained as follows: In the case that $e_{ms}^{a}{\left(P_{m}^{s}\right)}^{-1}<T$, the sensor node will be in energy-limited situation. The constraint can also be expended as:

(25) $$P_{m}^{s}\tau_{ms}+\left(P_{m}^{s}+g_{m}\right)\sum_{i=1}^{s-1}\tau_{mi}-\left\{e_{m1}^{a}+(s-1)Tg_{m}\right\}<0$$

In the case that $e_{ms}^{a}{\left(P_{m}^{s}\right)}^{-1}\geq T$, the sensor node has much energy for sensing in timeslot *s*. Similarly to that of the type-I sensor nodes, the sensing time of the sensor must be restricted to slot duration. So, the energy causality constraint for this case is simplified as $\tau_{ms}-T<0$. In the context of energy, the sensing time at the timeslot *s* is not affected by the sensing times of previous timeslots and will affect those of later timeslots.

Similarly to the case of the type-I sensor nodes, remaining energy of the type-II sensor nodes can be also manually controlled for various purposes. Let us denote $e_{ms}^{r}\in {ℜ}^{+}$ as the remaining energy of a sensor *m* at the end of timeslot *s*. Then the constraint for the remaining energy can be expressed as:

(26) $$e_{ms}^{r}=e_{m(s+1)}^{a}\geq e_{ms}^{r.th}$$

In terms of sensing time, this constraint for controlling the remaining energy is expressed by the following equation:

(27) $$\left(P_{m}^{s}+g_{m}\right)\sum_{i=1}^{s}\tau_{mi}-\left\{\left(e_{m1}^{a}+sTg_{m}\right)-e_{ms}^{r.th}\right\}<0$$

## 4. Throughput Maximization Formulation

In this section, we present how to evaluate the spectrum sensing performance of the activating cluster adopting multiple distributed EHSNs. In general, we can say that performance of the cluster of the SN is associated with the SACRN performance, which is represented by a considerable amount of data transferred over a licensed channel within a time duration. Recall the achievable throughput mentioned in Section 3.3. The total throughput of SACRN is evaluated over timeslots in a scheduling window, such that the optimal throughput will be a summation of optimal achievable throughput obtained in these all timeslots.

We first continue analyzing the achievable throughput of the SACRN as follows:

(28) $$R=\sum_{s=1}^{S}R_{s}=P_{0}\left\{\sum_{s=1}^{S}\frac{T-\tau_{s}}{T}(1-Q_{s}^{F})\right\}C_{0}$$

The expression can be further expanded as follows:

(29) $$R=\sum_{s=1}^{S}P_{0}C_{0}-\frac{1}{T}\sum_{s=1}^{S}P_{0}C_{0}\left\{TQ_{s}^{F}+\tau_{s}(1-Q_{s}^{F})\right\}$$

It is obvious that the first term in Equation (29) gives us a constant value. This is because the parameter *C*_0_ is a constant that depends on both bandwidth of the licensed channel and SNR presented at the secondary receiver in SACRN, whereas the parameter *P*_0_ is conditional upon the traffic of PU, but it can be approximately estimated over time. As a result, we can change the problem of throughput maximization to problem of the second term minimization of Equation (29). Thus, our problem is now formulated as follows:

(30) $$\left\{x_{vs}^{*},y_{vs}^{*}\right\}=\underset{x_{vs};y_{vs}}{\text{arg}\;\text{min}}U=\underset{x_{vs};y_{vs}}{\text{arg}\;\text{min}}\left\{\sum_{s=1}^{S}U_{s}=\sum_{s=1}^{S}PC\left\{TQ_{s}^{F}+\tau_{s}(1-Q_{s}^{F})\right\}\right\}$$

subject to:

(31) $$Q_{th}^{d}-Q_{s}^{D}\leq 0\;\;\;\forall s$$

(32) $$\tau_{ns}\leq min\left\{e_{ns}^{a}{\left(P_{n}^{r}\right)}^{-1},T\right\}\;\;\;\forall n,s$$

(33) $$\tau_{ms}\leq min\left\{e_{ms}^{a}{\left(P_{m}^{s}\right)}^{-1},T\right\}\;\;\;\forall m,s$$

(34) $$e_{vs}^{r}\geq e_{vs}^{r.th}\;\;\;\forall v\in \{n,m\},s$$

(35) $$x_{vs}\geq 0\;\;\;\forall v\in \{n,m\},s$$

(36) $$x_{vs}-y_{vs}\xi_{v}>0\;\;\;\forall x_{vs},y_{vs}$$

where $Q_{th}^{d}$ is the target global probability of detection that CSS among EHSNs needs to satisfy the constraint Equation (31) to protect the PU from illegal access of the SU in all timeslots of the scheduling window. The constraints Equations (32) and (33) represent energy-neutral operation for the type-I sensor nodes and the type-II sensor nodes of the cluster at every timeslot in the scheduling window, respectively, which are analyzed in the previous section. In addition, the constraint Equation (34) will control the amount of energy consumption in spectrum sensing at each timeslot, or the whole scheduling window, through controlling RE. Furthermore, the convexity of objective Function (30) is proved in Appendix A. For more details, see Appendix A.

Due to the complex diversity of setting constraints in the problem formulation, we could not directly solve the problem by any analyzed method, which may rely on the Karush-Kuhn-Tucker (KKT) condition as in the traditional method. Instead, we utilize an available optimization function called as *“fmincon()”* from the Matlab optimization toolbox to get $x_{vs}^{*},y_{vs}^{*}$ from Equation (30). The *“fmincon()”* is based on the active-set algorithm [17]. The active-set method is two-phase iteration method that provides an estimation of the set of active and inactive constraints at an optimal solution. In the first phase (the feasible phase), the objective is ignored while a feasible point is found for the constraints. In the second phase (the optimality phase), the objective is minimized by generating an iterative sequence of feasible points to converge to the solution while the feasibility is still maintained. Moreover, at each iteration in the second phase for finding a solution, the active set algorithm computes Lagrange multipliers λ associated with active constraints, such that the optimal solution is found if all λ ≥ 0 [18]. If the solution is not feasible, the algorithm will determine a new solution by adjusting step size parameter from the current solution such that the new solution is a feasible one. The active-set method is motivated by the fact that if the set of active and inactive inequality constraints at an optimal solution were known by the estimation, a solution could be found by solving a simpler equality constrained quadratic programming.

After getting $x_{vs}^{*},y_{vs}^{*}$ through numerical method based on “*fmincon()*” function, we can obtain optimal sensing time by using Equation (3) for sensor *v* of the cluster at the slot *s* that maximizes the throughput of sensor-aided cognitive radio network where *s* = 1, …, *S*, and *v* = 1, …, *V*.

## 5. Performance Evaluations

In this section, we present simulation results to show the achievable throughput with SACRN under different EHSN energy harvesting scenarios. Throughput of SACRN is examined over 12 timeslots, and various WS is set up as *S* ∈ [1, 2, 3, 4, 6, 12] to fit in the 12-timeslots. Recall that, due to different locations of EHSNs, the received SNR of the primary signal, the available energy in the battery, and the energy harvesting rate at an EHSN node can differ from those at other nodes. For simulations, the SNR at an EHSN node is generated by an exponential distribution with the mean μ. For energy harvesting rate of a sensor node, a value of *g_min_* is generated randomly in (0.2, 0.6), and is set as constant for in the window. The main simulation parameters for the whole SACRN are given in Table 1.

**Table 1:** Main simulation parameters.

Parameters	Description	Value/Metric
*T*	slot duration	10 ms
*C*_0_	maximal throughput of channel	10 kb/slot
*P*_0_	probability of free slot	0.4
*f_s_*	sampling frequency of PU signal	200 kHz
μ	mean of SRN distribution	−10 dB
$P_{v}^{s}$	sensing power of sensor nodes	15 mW
$Q_{th}^{d}$	probability of detection threshold	0.9

For the first experiment, we consider a cluster where four EHSN nodes with different SNRs and energy harvesting rates exist, and no control of remaining energy is considered. Here, “no control of remaining energy” means that the constraint expressed by Equation (34) is not considered in the experiment.

**Figure 4:**
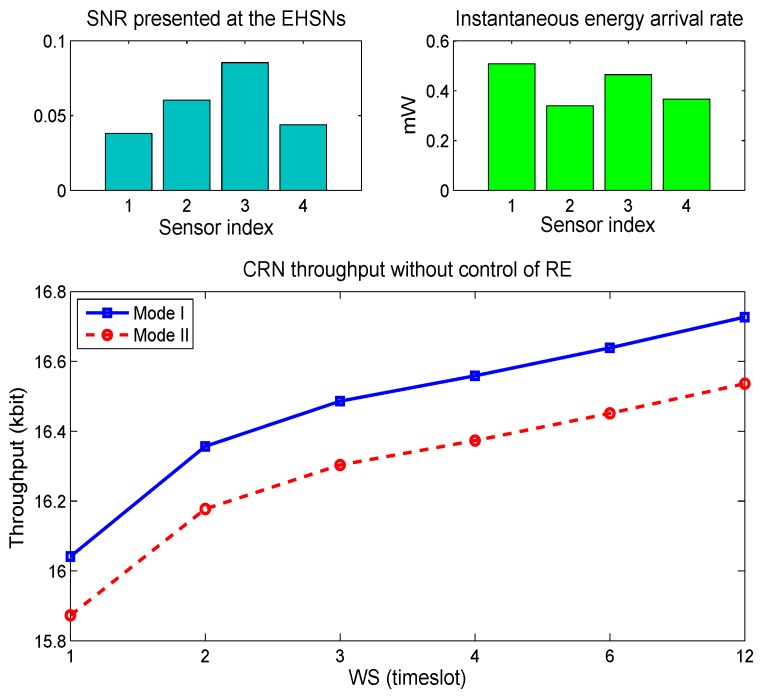
Throughput of SACRN according to two operation modes when the control of remaining energy is not considered.

Initial energy for EHSNs at the beginning of the scheduling window, which means the available energy at the beginning of the first timeslot, is set to 20 µJ. Further we consider the two modes. In the Mode I, all four sensor nodes operate as type-I node. On the other hand in the Mode II, all four sensor nodes operate as type-II node. Figure 4 shows the throughput of the SACRN during 12 timeslots according to various sizes of scheduling windows. For both of mode operations, the throughput increases as the WS increases. The SACRN also get more throughput when it is operated in Mode I. That is due to the fact that EHSN nodes under Mode II can harvest less energy since they do not harvest energy in the sensing duration. Also, Figure 4 shows that the throughput of SACRN comes to a minimum value when the WS is one. The scheduling when WS = 1 is quite similar to the “Myopic scheme” [4], where a single secondary user only tries to take immediate throughput or reward at a current timeslot without considering other future timeslots for long-term throughput.

**Figure 5:**
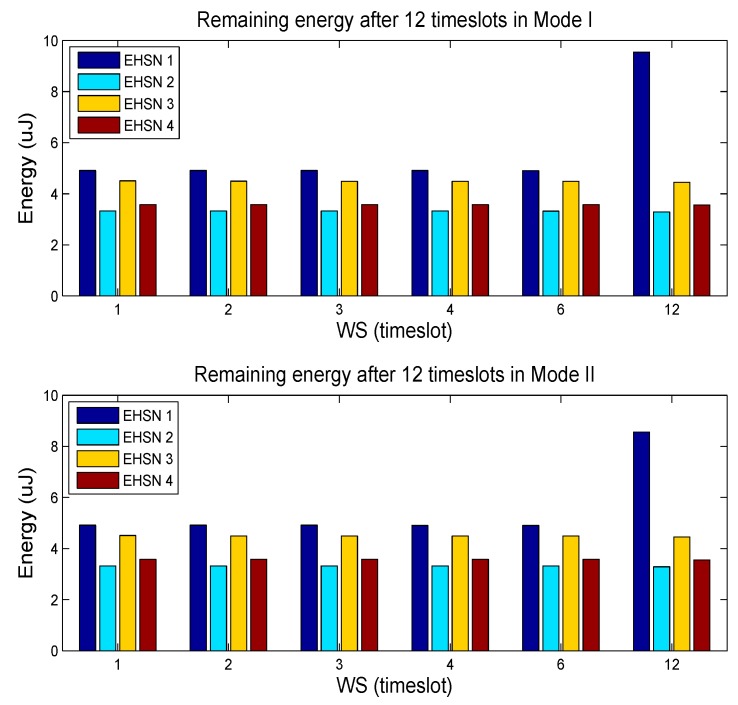
Remaining energy of EHSN nodes after 12 timeslot scheduling when the control of remaining energy is not considered.

Figure 5 shows the remaining energy of four sensor nodes according to different window sizes in the Mode I and Mode II respectively. It is observed that the remaining energy of all EHSN nodes when *WS* = 12 differs from those of other cases. Since the sensor node 1 has higher energy harvesting rates and low SNR, the sensor node 1 would not consume as much energy for spectrum sensing. That is, sensor nodes with higher SNR participated in the spectrum sensing more while sensor nodes with lower SNR participated in the spectrum sensing less. Therefore, the remaining of sensor nodes 1 is quite large while the remaining energies of other sensor nodes are low.

**Figure 6:**
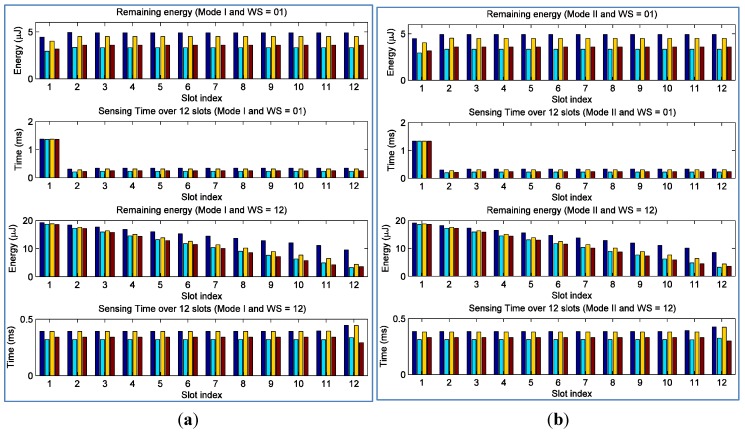
Remaining energy and sensing time of each sensor at each timeslot when WS = 1 and WS = 12, and the control of remaining energy is not considered; (**a**) in the mode I (**b**) in the mode II.

Figure 6 shows the details of remaining energy and sensing time of four EHSN nodes over 12 timeslots for both operation modes when WS = 1 and WS = 12, respectively. When WS = 1, it is clearly observed that all EHSN nodes tried to independently perform CSS in each timeslot without consideration of other timeslots. For example, in the first timeslot EHSN nodes spent much energy for sensing to obtain much throughput, which resulted in less remaining energy. After that, these sensor nodes only relied on harvested energy for sensing in subsequent timeslots. When WS = 12, on the other hand, energy for spectrum sensing was distributed in a better way. The remaining energy at some sensor nodes might reduce gradually as timeslot goes and the remaining energy of sensor node 1 is still high due to the low SNR. Furthermore, the sensing times of EHSN nodes in Mode I are longer than those in Mode II. This decreases the global probability of false alarm and increases throughput while maintaining the global probability of detection above the given threshold.

**Figure 7:**
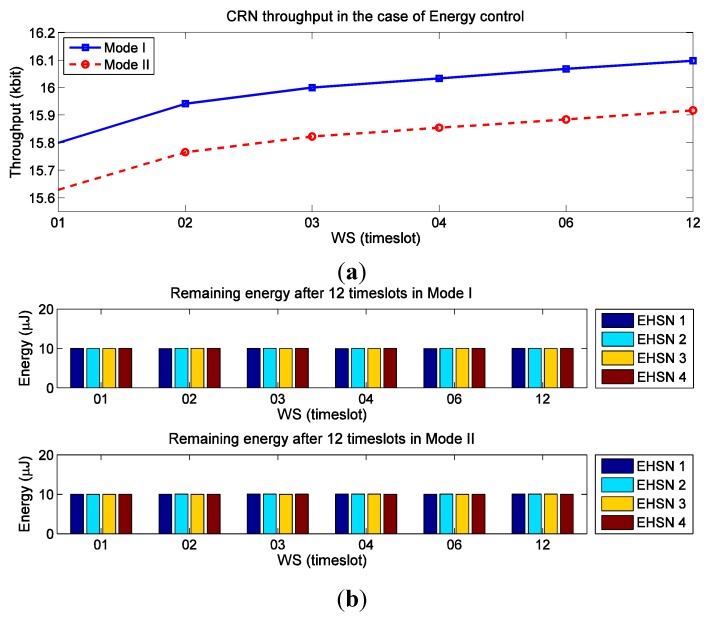
Throughput of SACRN and remaining energy of each EHSN node in two modes when remaining energy is controlled. (**a**) The achievable throughput of SACRN under the control of remaining energy; (**b**) The remaining energy of EHSN nodes under the control of remaining energy.

For the second experiment, we utilize the same simulation parameters used in the first experiment, but we consider the control of remaining energy, which is equivalent to the constraint Equation (34). That is, remaining energy of all sensor nodes after 12-timeslot scheduling should be larger than 10 μJ. In this case, throughput of SACRN, remaining energy, and sensing time of each EHSN node are observed. At first, Figure 7a shows the achievable throughput of SACRN under the control of remaining energy. When Figure 7a is compared with Figure 4, we can observe that, due to limit of energy consumption on spectrum sensing, the throughput when the control of remaining energy is used is less than that when the control of remaining energy is not considered. Figure 7b shows that the remaining energies of EHSN nodes after 12-timeslot scheduling are well kept at 10 μJ as required.

**Figure 8:**
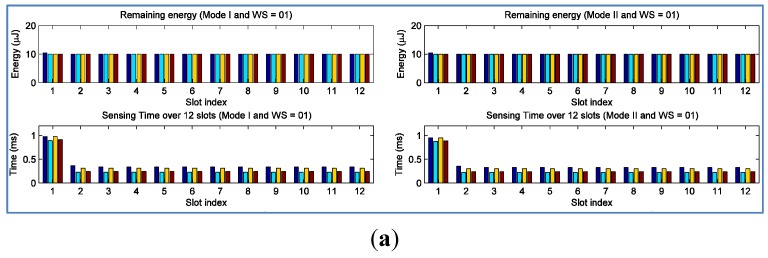

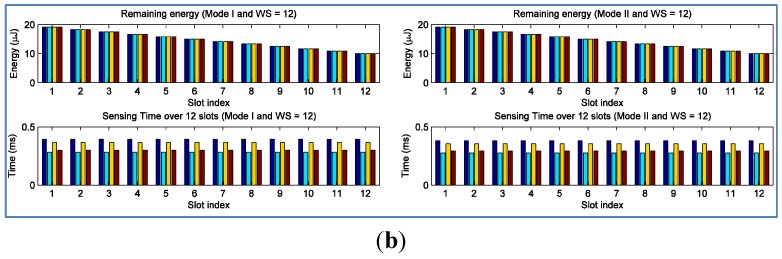
Remaining and sensing time of EHSNs over 12 timeslots when remaining energy is controlled. (**a**) Remaining energy and sensing time of EHSNs in Mode I and Mode II when WS = 1; (**b**) Remaining energy and sensing time of EHSNs in Mode I and Mode II when WS = 12.

Figure 8 shows remaining energy and sensing time of EHSNs over timeslots, similarly to the case of no control of remaining energy shown in Figure 6. Figure 8a shows remaining energy in Mode I and Mode II when WS = 1. When WS = 1, all EHSNs tried to maximize throughput in each slot such that they consumed much energy for spectrum sensing at the first timeslot, and remaining energy of all EHSNs was immediately reduced to 10 μJ. Figure 8b shows that energy can be used in better way when WS = 12. When WS = 12, remaining energy decreases gradually as timeslot goes, which implies that energy is shared reasonably and distributed among timeslots within the window. Therefore, we can achieve more throughput of SACRN when WS = 12. Figure 8 also shows that the sensing time of EHSNs in Mode I is longer than that in Mode II due to more harvesting energy, similarly to observation of the first experiment.

In the last experiment, we consider SACRN in which three type-I sensor nodes (*N* = 3) and three type-II sensor nodes (*M* = 3) coexist. Initial energy of all EHSNs was also set to 20 μJ. Four remaining energy constraints after 12 timeslots are considered, and are given by 15 μJ, 17 μJ, 19 μJ and 21 μJ, respectively.

**Figure 9:**
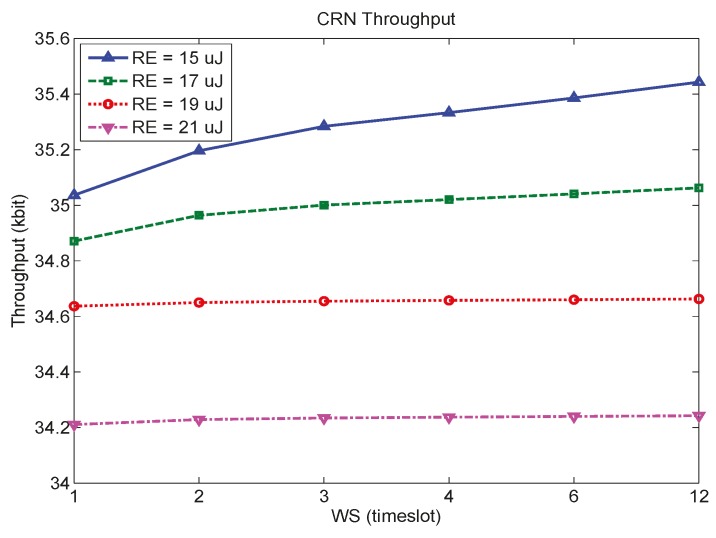
Throughput of SACRN according to WS for different remaining energy constraints.

Figure 9 shows the throughput of SACRN according to WS for different remaining energy constraints. It is observed that SACRN achieves higher throughput when less remaining energy constraint is required since more energy can be spent for spectrum sensing, which results in lower false alarm probability and consequently more throughput. Figure 9 also show that the effect of WS on throughput is more significant when less remaining energy constraint is required. When remaining energy constraint is given as 21 μJ, which is higher than the initial energy, EHSN nodes try to accumulate energy from environment rather than spending energy for spectrum sensing such that the throughput is little improved by increasing WS.

## 6. Conclusions

In this paper, cognitive radio technology and green wireless systems are discussed with the objective of maximizing total achievable throughput of SACRN under the limitation of energy supply and energy harvesting scenarios. We first consider two operation modes for different types of EHSNs from ambient energy harvesting, and then investigate the energy constraints for spectrum sensing operations in a scheduling window. We also examine aspects of remaining energy in the scheduling window to take the control of sensing energy consumption into account. We present the convex function formulation-based sensor scheduling method under several energy scenarios in the energy harvesting sensor network to maximize the throughput. The simulation results indicate that energy harvesting sensor nodes need to be scheduled over more timeslots for better throughput in an energy harvesting scenario. The size of scheduling window may depend on practical conditions, such as computational complexity and computing capability of devices. However, the SACRN can achieve better throughput when factors that affect sensor scheduling and energy distribution, such as energy harvesting rate can be estimated.

## Appendix A

To prove the convexity of objective Function (30), let’s begin to show the first and second derivatives of Q function as follows:

(A1) $$\frac{dQ(x)}{dx}=-\frac{1}{\sqrt{2\pi }}exp\left(-\frac{x^{2}}{2}\right)<0$$

(A2) $$\frac{d^{2}Q(x)}{dx^{2}}=\frac{x}{\sqrt{2\pi }}exp\left(-\frac{x^{2}}{2}\right)$$

Using Equation (3), we can obtain the first and second derivatives of τ_*vs*_ with respect to *x_vs_* and *y_vs_* as follows:

(A3) $$\frac{\partial \tau_{vs}}{\partial x_{vs}}=\frac{2\left(x_{vs}-y_{vs}\xi_{v}\right)}{\delta_{v}^{2}}>0$$

(A4) $$\frac{\partial^{2}\tau_{vs}}{\partial x_{vs}^{2}}=\frac{2}{\delta_{v}^{2}}>0$$

(A5) $$\frac{\partial \tau_{vs}}{\partial y_{vs}}=\frac{-2\xi_{vs}\left(x_{vs}-y_{vs}\xi_{v}\right)}{\delta_{v}^{2}}=-\xi_{v}\frac{\partial \tau_{vs}}{\partial x_{vs}}<0$$

(A6) $$\frac{\partial^{2}\tau_{vs}}{\partial y_{vs}^{2}}=\frac{2\xi_{s}^{2}}{\delta_{s}^{2}}>0$$

Using Equation (4), we can obtain the first and second derivatives of $Q_{s}^{F}$ with respect to *x_vs_* as follows:

(A7) $$\frac{\partial Q_{s}^{F}}{\partial x_{zs}}=\frac{dQ(x_{zs})}{dx_{zs}}\prod_{z\neq v}\left[1-Q(x_{vs})\right]<0$$

(A8) $$\frac{\partial^{2}Q_{s}^{F}}{\partial x_{zs}^{2}}=\frac{d^{2}Q(x_{zs})}{dx_{zs}^{2}}\prod_{z\neq v}\left[1-Q(x_{vs})\right]$$

Similarly, we can obtain $\frac{\partial Q_{s}^{D}}{\partial y_{zs}}<0$ and $\frac{\partial^{2}Q_{s}^{D}}{\partial y_{zs}^{2}}<0$ using Equation (5).

Therefore, we can obtain the first and second derivatives of *U* with respect to *y_vs_* and *x_vs_* as follows:

(A9) $$\frac{\partial U}{\partial y_{vs}}=P_{0}C_{0}\left(1-Q_{s}^{F}\right)\frac{\partial \tau_{s}}{\partial y_{vs}}>0$$

(A10) $$\frac{\partial^{2}U}{\partial y_{vs}^{2}}=P_{0}C_{0}\left(1-Q_{s}^{F}\right)\frac{\partial^{2}\tau_{s}}{\partial y_{vs}^{2}}>0\;\;\forall x_{vs}$$

(A11) $$\frac{\partial U}{\partial x_{vs}}=P_{0}C_{0}\left\{(T-\tau_{s})\frac{\partial Q_{s}^{F}}{\partial x_{vs}}+(1-Q_{s}^{F})\frac{\partial \tau_{s}}{\partial x_{vs}}\right\}$$

(A12) $$\frac{\partial^{2}U}{\partial x_{vs}^{2}}=P_{0}C_{0}\{-2\frac{\partial \tau_{s}}{\partial x_{vs}}\frac{\partial Q_{s}^{F}}{\partial x_{vs}}+(T-\tau_{s})\frac{\partial^{2}Q_{s}^{F}}{\partial x_{vs}^{2}}+(1-Q_{s}^{F})\frac{\partial^{2}(\tau_{s})}{\partial x_{vs}^{2}}\}$$

From Equations (A9) and (A10), therefore we know that *U* is a decreasing and convex function with respect to *y_vs_*. The second derivative of *U* with respect to *x_vs_* is always positive if the second derivative of $Q_{s}^{F}$ with respect to *x_vs_* is positive. Since $P_{vs}^{f}$ is always given less than 0.5, we get *x_vs_* > 0. Since *x_vs_* > 0, the second derivative of $Q_{s}^{F}$ with respect to *x_vs_* is always positive. Therefore, the second derivative of *U* with respect to *x_vs_* is always positive. Therefore, *U* is a convex function of *x_vs_*.
